# Efficiency of using electric toothbrush as an alternative to a tuning fork for artificial buzz pollination is independent of instrument buzzing frequency

**DOI:** 10.1186/s12898-020-00278-7

**Published:** 2020-02-10

**Authors:** Mandeep Tayal, Jesus Chavana, Rupesh R. Kariyat

**Affiliations:** Department of Biology, The University of Texas Rio Grande Valley, Edinburg, TX 78541 USA

**Keywords:** Buzz pollination, Pollen extraction, Solanum, Tuning fork, Electric toothbrush, Frequency

## Abstract

**Background:**

Breeding programs and research activities where artificial buzz-pollinations are required to have primarily relied upon using tuning forks, and bumble bees. However, these methods can be expensive, unreliable, and inefficient. To find an alternative, we tested the efficiency of pollen collection using electric toothbrushes and compared it with tuning forks at three vibration frequencies—low, medium, and high and two extraction times at 3 s and 16 s- from two buzz—pollinated species (*Solanum lycopersicum* and *Solanum elaeagnifolium*).

**Results:**

Our results show that species, and extraction time significantly influenced pollen extraction, while there were no significant differences for the different vibration frequencies and more importantly, the use of a toothbrush over tuning fork. More pollen was extracted from *S. elaeagnifolium* when compared to *S. lycopersicum*, and at longer buzzing time regardless of the instrument used.

**Conclusions:**

Our results suggest that electric toothbrushes can be a viable and inexpensive alternative to tuning forks, and regardless of the instrument used and buzzing frequency, length of buzzing time is also critical in pollen extraction.

## Background

In another wonderful example of convergent evolution, it is estimated that around 6% of flowering plants, comprising species from multiple plant families, are primarily buzz-pollinated [1, 2]. Among these species, the most common anther type is poricidal, where pollen grains tend to be stored inside non-dehiscent anther tubes with small pores at the tip [3]. Concealing pollen grains inside poricidal anthers conserves pollen, and has also led to specialized pollinators, commonly known as buzz pollinators. More interestingly, these pollinators mainly include bumble bees (*Bombus* spp.), carpenter bees (*Xylocopa* spp.), and sweat bees (*Lasioglossum* spp.) among others, but not honeybees (*Apis* spp.) [4]. Unlike other insect pollinators (e.g., Lepidoptera), buzz pollinators produce floral vibrations using their thoracic muscles and use their other body parts including mandibles, head and abdomen to release the pollen from these anthers [1, 5–9], an ability confined to a few insect genera. Although studies on ecology and evolutionary biology of buzz pollination have been carried out for more than a century [10], the biomechanics, pollinator physiology and behavior in relation to buzzing have only recently gained an increased interest [1, 11, 12].

Solanaceae is one of the major plant families that are predominantly buzz-pollinated. They include crops such as tomato (*Solanum lycopersicum*), peppers (*Capsicum* spp.), eggplant (*Solanum melongena*), and weeds such as horsenettle (*Solanum carolinense*), buffalo bur (*Solanum rostrum*) and silverleaf nightshade (*Solanum elaeagnifolium*) to name a few. Equally important for crop husbandry purposes and ecological research, pollination experiments in these species essentially require the manipulation of poricidal anthers to collect pollen. For example, both *S. carolinense* and *S. elaeagnifolium* are obligate outcrossing species with gametophytic self-incompatibility (SI) but will undergo selfing under certain circumstances such as lack of foreign pollen and increase in floral age [13], and any manipulative empirical studies on these require pollen extraction at our convenience. In cultivated species such as *S. lycopersicum* and *S. melongena*, most breeding programs and variety trials require the extraction and analysis of pollen, and subsequent artificial pollination [14, 15]. Previous studies shows that synthetic stimuli [16], vibrations produced by transducers [17] and tuning forks [4, 18, 19], can be used in artificial pollen extraction. Among these, tuning forks are commonly employed in most of studies for pollen extraction. For such extractions, the tuning fork is allowed to vibrate and held close to the anthers, thereby releasing the pollen, which is collected into a tube for further use [4]. However, tuning forks can be expensive, hard to find with right frequency for field experiments, and more importantly, tend to break if struck hard before initiating the vibration cycle (personal observation). Since a significant part of ecological research is done in field which limits the access to find appropriate replacement for tuning forks in a timely fashion, this can severely hamper the experiments.

To find an alternative for tuning forks, we tested the pollen extraction efficiency of electric toothbrushes, which are cheaper, easier to find, and much more reliable. However, pollen extraction through buzzing could also be affected by species variation, time of buzzing and also by the frequency of vibrations. For example, it has been shown that vibrations at high frequencies (450–1000 Hz) ejects more pollen as compared to the low frequency (100–400 Hz) vibrations [17]. To account for these factors, we carried out an experiment where we collected pollen from two Solanaceous species, an invasive weed Silverleaf nightshade (*S. elaeagnifolium)*, and tomato (*S. lycopersicon*). In addition, we tested the efficiency of pollen removal at multiple buzzing frequencies for both electric toothbrushes and tuning forks, at two time intervals. Since floral vibrations produced by bees are substrate-borne vibrations affected by time and frequency [1], we hypothesized that both instruments would extract similar amounts of pollen. In addition, we also hypothesized that both frequency and time of collection would significantly affect pollen extraction, also affected by the plant species.

## Results

**Fig. 1:**
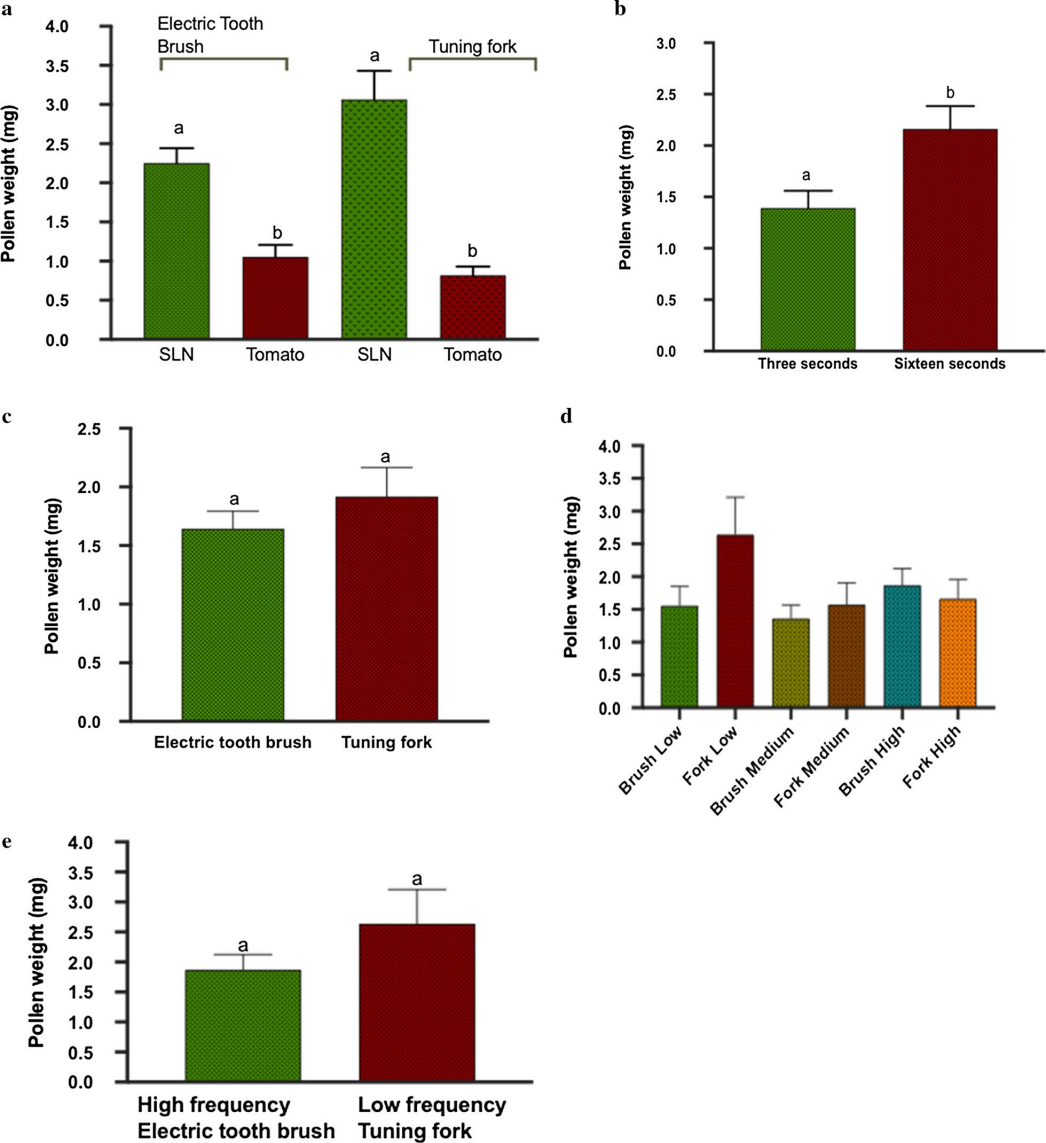
The amount of pollen extracted in different treatments. Post hoc Tukey’s test (p < 0.05) for pollen extraction from **a** Tomato and Silverleaf nightshade, **b** different time intervals, **c** electric toothbrush and tuning fork at **d** different instrument vibrations frequency levels and **e** low frequency tuning fork and high frequency electric toothbrush. Means followed by same letters are not significantly different (p < 0.05). Different letters show means are significantly different (p < 0.05)

We found significant differences among treatments for pollen extraction (Table 1A). Among the factors, we found that plant species, and length of vibration time were statistically significant. We extracted significantly more pollen from *S. elaeagnifolium* when compared to *S. lycopersicum* (Fig. 1a), and among time intervals, 16 s of vibration significantly extracted more pollen when compared to 3 s (Fig. 1b). More interestingly, we found that there was no significant difference between the use of tuning fork and electric toothbrush even at multiple time intervals and vibration frequencies for these two species (Fig. 1c). We also found that different frequency levels of both instrument vibrations did not affect pollen extraction (Fig. 1d). Even the extreme comparison of high-frequency electric toothbrush with low frequency tuning fork extracted almost equal amounts of pollen (Fig. 1e). Among the interactions, only instrument X species was significant, where using an electric toothbrush on *S. elaeagnifolium* extracted more pollen (Table 1B) than electric tooth brush and tuning fork on *S. lycopersicum*, and tuning fork on *S. elaeagnifolium* extracted more pollen than electric tooth brush and tuning fork on *S. lycopersicum*, although the instrument difference did not affect pollen extraction within the species.

**Table 1:** ANOVA for the pollen extraction

Source	DF	F ratio	Prob > F
Panel A			
Model	11	10.7507	
Error	84		
C. Total	95		< 0.0001*
Panel B			
Instrument	1	0.6431	0.4249
Species	1	87.5024	< 0.0001*
Time	1	18.4352	< 0.0001*
Frequency	2	1.4225	0.2469
Instrument * species	1	5.3229	0.0235*
Species * time	1	0.6864	0.4097
Time * frequency	2	1.1406	0.3245
Species * time * frequency	2	1.0708	0.3474

Statistical analysis (ANOVA) of pollen extraction from *S. elaeagnifolium* and *S. lycopersicum* using tuning fork and electric toothbrush at different frequencies for 3 and 16 s time intervals

## Discussion

The major take away from our results is that we didn’t find any significant difference in the amount of pollen collected using an electric brush over a tuning fork, which was our primary factor of interest. As tuning forks are expensive (cost ranges $8–$11 each), less durable and difficult to replace in the field, our results clearly show that they can be substituted with an inexpensive (cost ranges $4–$6 each), and durable electric toothbrush. In addition, our results clearly show that the species and buzzing time are significant factors in pollen extraction in artificial buzzing regardless of the vibration frequency and type of instrument. The greater the buzzing duration, the more pollen is extracted, and this result aligns with the previous work that showed a positive correlation of high amplitude and buzzing duration on pollen ejection in *S. rostratum* [16], a species with similar floral traits as *S. elaeagnifolium* and *S. lycopersicum*. This is primarily because with longer buzzing time, vibrations are generated and transmitted for a longer time and consequently, release more pollen. However, the discrepancy found between claimed and observed toothbrush frequency restricted us in comparative frequency analysis between both instruments. Between the two species tested, we extracted the higher amount of pollen in *S. elaeagnifolium* as compared to *S. lycopersicum*. The presence of more pollen in *S. elaeagnifolium* might also contribute to high fruit set [20] and colonization success of this weed species. Our results also showed no differences in pollen amount extracted among different frequency levels. This was somewhat surprising because, recently, it has been found that larger bees that generate high floral vibration frequencies extract more pollen when compared to small bees in a given foraging effort [11], also suggesting that there may be additional effects of pollinator-specific buzzing that affect pollen removal [1].

The Solanaceae plant family is a model for studying SI and the species that exhibit it tend to be obligate outcrossers, and in some cases, SI breaks down with floral age [13] leading to selfing, and consequently inbreeding depression, [21] which plays a significant role in the evolution of mating systems [22]. Most studies on inbreeding and/or genetic variation and their effects on fitness traits require pollen extractions, pollen trait measurements, and controlled pollinations [23]. In the case of tomatoes and other economically important crops, breeding programs also require the use of such methods for pollen extraction and subsequent selection studies. Bumble bees and tuning forks have traditionally been employed for these respectively, but here we show that cheap and easily available electric toothbrushes can be used as a viable alternative to these methods, producing similar results. However, one concern we had was for *S. elaeagnifolium*, the flowers were collected from the field early in the morning, assuming they weren’t pollinated yet (personal observations). Ideally, we would want to grow them also as an experimental population in controlled conditions. Future research should also involve comparative studies on insect pollinators and artificial methods to tease out the differences in the characteristics that separate them, and their consequences on pollen removal and plant fitness. Although a disparity in manually calculated frequency and software-calculated frequency was observed in electric toothbrushes, it didn’t affect our experimental results showing pollen collection is independent of buzzing frequencies in artificial buzzing.

## Conclusions

Our results show that electric toothbrush can be used as a viable alternative to tuning fork in artificial buzz pollination. In addition, our study also indicate that more research in buzz pollination should be focused on how species variation and duration of buzzing affect pollen extraction efficiency, areas we are currently exploring.

## Materials and methods

### Study species

For the experiments detailed below, we used two buzz-pollinated *Solanum* species, i.e. silverleaf nightshade (*S. elaeagnifolium*) and tomato (*S. lycopersicum)*. Silverleaf nightshade is a worldwide invasive perennial weed, native to the southwestern United States and Mexico [24]. The flowers are usually blue lilac in color, nectar-less, hermaphrodite and have poricidal anthers mostly visited by buzz pollinators (carpenter bees: *Xylocopa* spp., bumble bees: *Bombus* spp., sweat bee: *Lasioglossum* spp.) for pollen transfer and reproduction success [20]. It acts as ruderal, colonizes disturbed sites and is also toxic to livestock [24]. However, tomato is an herbaceous, economically important agricultural crop widely cultivated throughout the world. The flowers are nectar-less, yellow in color and anthers are laterally bound together with pore-like openings at the apical end [25]. Flower agitation either by wind or natural pollinators (bumble bee, sweat bee, carpenter bee) is crucial for pollen removal [26].

### Plant material

The plant species used in the study were either grown in controlled conditions (*S. lycopersicum*) or sampled (*S. elaeagnifolium*) from the local native population. We used F1 tomato hybrid seeds (Variety: Valley Girl, Product ID 741, Johnny’s Selected Seeds, ME, USA) sown in growth media (Sunshine professional growing mix: Sun Gro Horticulture Canada Ltd., MA, USA) in the plastic trays (51.435 cm * 25.4 cm) and covered with thin transparent film to maintain optimum temperature of 27 °C for germination. At 2–4 leaf stage, the seedlings were repotted individually to bigger pots (15.24 cm diameter) and kept in greenhouse at 25 °C and 65% RH. Plant nutrient requirements were met by applying OMRI (Organic Material Review Institute, OR, USA) listed organic fish emulsion fertilizer (NPK 5:1:1, Alaska Fish Fertilizer, Pennington Seed, Inc., GA, USA) once every 2 weeks. Plant growth and health were maintained until flowering and plants were ready for experiment.

On the other hand, for *S. elaeagnifolium*, we used flowers from multiple native populations in the City of Edinburg and Mission, Texas (26° 18′ 25.8″ N 98° 12′ 10.9″ W; 26° 11′ 35.6″ N 98° 19′ 11.3″ W). In synchronization to the tomato flowers, we selected silverleaf nightshade plants with at least 5 fully opened new flowers, and the plants were cut using a pair of pruning shears. After collecting the plants with flowers, they were immersed in water up to 7–8 cm and were immediately brought back to the lab. The plant sampling was done early in the morning before pollinators visits to avoid any prior floral visits (personal observations).

### Instruments and treatments

Our experimental design was to examine the effects of buzzing instrument, buzzing time, and frequency differences on pollen removal from these two species. To accomplish that we used tuning forks (Tuning fork aluminum alloy, Lot No: 3200-x, Ward’s Science, New York, USA) cost ranges $8–$11 each of different frequencies, i.e. low (256 Hertz (Hz), medium (320 Hz) and high (512 Hz). We also used the electric toothbrushes, which cost ranging from $4 to $6 each of different strokes i.e. 14,000/min (233/s or 233 Hz) (Oral-B 3d White Action Power Toothbrush), 20,000/min (333/s or 333 Hz) (Colgate 360 powered toothbrush, Colgate Co. Pvt. Ltd.) and 30,000/min (500/s or 500 Hz) (Vivid Sonic Clean toothbrush) We used a digital acoustic recorder (Tascam DR-100 MK-III: TEAC America, Inc., CA, USA) to record each of their vibration frequencies (see Additional files 1, 2, 3) and then analyzed the files in Audacity v. 2.1.3 (https://sourceforge.net/projects/audacity/) by examining the spectrogram using ‘Spectrogram’ function (FFT = 8192 Hz, Hamming window). We found a different range of frequencies than those advertised (Additional file 7). The tuning fork vibrational frequencies (see Additional files 4, 5, 6) were also verified in this software, but were found to be consistent with the advertised frequencies (Additional file 7).

### Detailed methodology

**Fig. 2:**
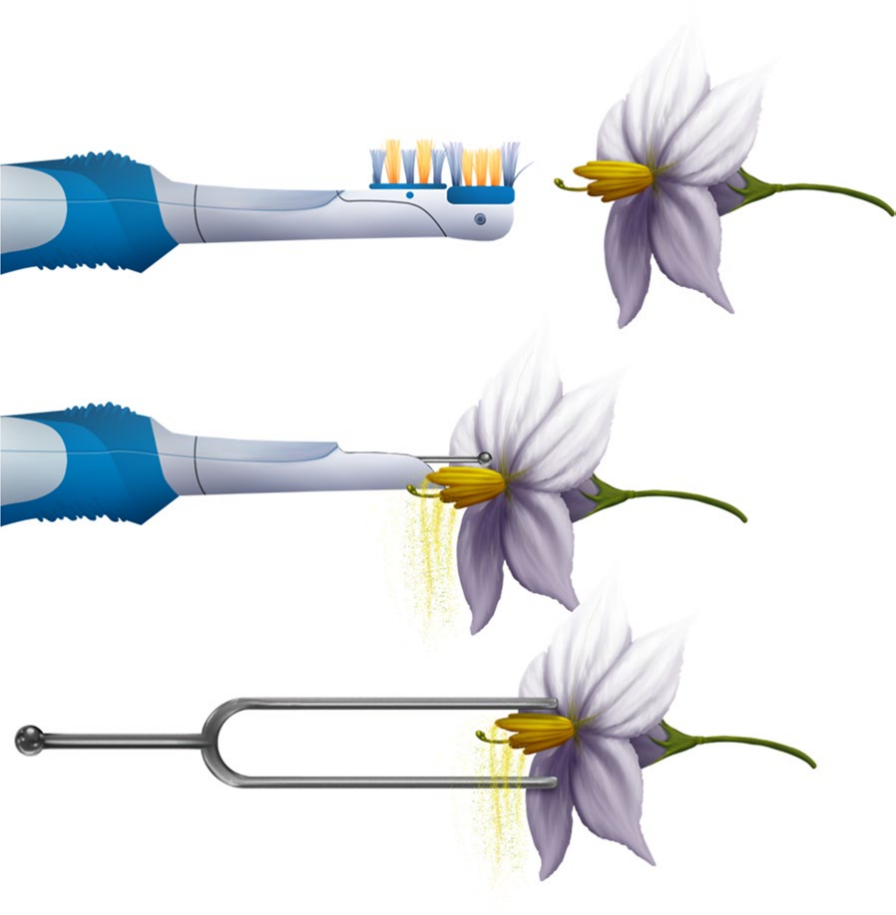
Schematic representing artificial buzz pollination using a toothbrush and a tuning fork. The bristle head of the toothbrush is removed, and the metal nub is held near the anthers to vibrate. In the case of tuning fork, the prongs are held over the anthers or near them. Cartoon by Annette Diaz, University of Texas Rio Grande Valley

As mentioned above, the *S. elaeagnifolium* plants were sampled and brought to the lab on each day of the experiment. *S. lycopersicum* plants with newly opened flowers were moved from greenhouse to the lab. Both species were tested in tandem. At first, the tuning fork of low frequency (259 Hz) was used for 3 s to extract the pollen. For this, the tuning fork was hit on the lab counter top, and then it was brought close to the flower without making contact. The resulting pollen was collected in 0.5 ml PCR tubes (Pryme PCR: Midwest Scientific, MO, USA). The same procedure was repeated for same frequency but for a different time (16 s) interval. For the other half of the plants, we followed the same methodology, except that an electric brush was used instead of the tuning fork. The bristle head of the brush was removed, and anthers were vibrated by bringing metal nub near to the anthers. The same procedure was repeated for other frequencies, i.e. medium and high in both species. To collect enough pollen for better weight measurement, we pooled pollen from three flowers for each treatment, and then weighed the sample. An empty 0.5 ml tube was weighed and the PCR tubes containing pollen were weighed to get pollen weight. Weight measurements were carried out using an advanced digital balance (Accuris *Series Dx*, Model: W3101A-220, Benchmark Scientific, NJ USA). A schematic of the experiment is detailed in Fig. 2.

### Statistical analysis

Due to the non-normal nature of the data set, the raw data were transformed using Squareroot + 1transformation prior to analysis of variance. We used the weight of pollen collected as our response variable and instrument, species, time, and frequency, and their interactions as our fixed factors. Means were separated and pairwise comparisons were carried out using the post hoc Tukey tests at p < 0.05. All analyses were carried out using the statistical software JMP (Statistical Analysis Software (SAS) Institute, Cary, NC, USA).

## Supplementary information

**Additional file 1.** Low frequency vibrational sound produced by an electric toothbrush during an experiment.

**Additional file 2.** Medium frequency vibrational sound produced by an electric toothbrush during an experiment.

**Additional file 3.** High frequency vibrational sound produced by an electric toothbrush during an experiment.

**Additional file 4.** Low frequency vibrational sound produced by a tuning fork during an experiment.

**Additional file 5.** Medium frequency vibrational sound produced by a tuning fork during an experiment.

**Additional file 6.** High frequency vibrational sound produced by a tuning fork during an experiment.

**Additional file 7: Table S1.** Comparison of manual and actual values of different frequency levels of tuning fork and electric toothbrush analyzed in Audacity software using Digital Acoustic recorder.

## Data Availability

The data sets supporting the results of this article are available in the Dryad Digita Repository [27], 10.5061/dryad.rd72rt2.

## Abbreviations

SI: Self incompatability
OMRI: Organic Material Review Institute
Hz: Hertz
TEAC: Tokyo Electro-Acoustic Company
PCR: Polymerase chain reaction
JMP: Jump (Statistical Software)
