# Supplementary material for: Efficiency of using electric toothbrush as an alternative to a tuning fork for artificial buzz pollination is independent of instrument buzzing frequency
Source: BMC Ecol. 2020 Feb 10;20:8. doi: 10.1186/s12898-020-00278-7 (PMC7008546; doi:10.1186/s12898-020-00278-7)
Supplement: Supplementary file 7 — Additional file 7: Table S1. Comparison of manual and actual values of different frequency levels of tuning fork and electric toothbrush analyzed in Audacity software using Digital Acoustic recorder. [file 12898_2020_278_MOESM7_ESM.docx]

Additional Table S1. Comparison of manual and actual values of different frequency levels of tuning fork and electric toothbrush analyzed in Audacity software using Digital Acoustic recorder.

| ***Instrument type*** | ***Frequency level*** | ***Expected/Manual frequency (Hz)*** | ***Actual/Calculated frequency (Hz)*** |
| --- | --- | --- | --- |
| **Tuning fork** | Low | 256 | 259 |
|  | Medium | 320 | 320 |
|  | High | 512 | 513 |
| **Electric toothbrush** | Low | 233 | 137 |
|  | Medium | 333 | 173 |
|  | High | 500 | 249 |
